# Wavelet Texture Descriptor for Steel Surface Defect Classification

**DOI:** 10.3390/ma17235873

**Published:** 2024-11-29

**Authors:** Djilani Belila, Belal Khaldi, Oussama Aiadi

**Affiliations:** 1 Department of Computer Science and Information Technologies, University of Kasdi Merbah, Ouargla 30000, Algeria; 2 Laboratoire d’Intelligence Artificiel et des Technologies de l’Information (LINATI), Ouargla 30000, Algeria

**Keywords:** image classification, steel surface defect, multiscale wavelet decomposition, texture analysis, wavelet texture descriptor, industry image analysis

## Abstract

The accurate and efficient classification of steel surface defects is critical for ensuring product quality and minimizing production costs. This paper proposes a novel method based on wavelet transform and texture descriptors for the robust and precise classification of steel surface defects. By leveraging the multiscale analysis capabilities of wavelet transforms, our method extracts both broad and fine-grained textural features. It involves decomposing images using multi-level wavelet transforms, extracting a series set of statistical and textural features from the resulting coefficients, and employing Recursive Feature Elimination (RFE) to select the most discriminative features. A comprehensive series of experiments was conducted on two datasets, NEU-CLS and X-SDD, to evaluate the proposed method. The results highlight the effectiveness of the method in accurately classifying steel surface defects, outperforming the state-of-the-art techniques. Our method achieved an accuracy of 99.67% for the NEU-CLS dataset and 98.24% for the X-SDD dataset. Furthermore, we demonstrate the robustness of our method in scenarios with limited data, maintaining high accuracy, making it well-suited for practical industrial applications where obtaining large datasets can be challenging.

## 1. Introduction

The integrity of material surfaces, particularly in steel manufacturing, is paramount for ensuring the quality and safety of products critical to the aerospace, automotive, and precision machinery industries. Defects on these surfaces, if undetected or improperly classified, can lead to significant compromises in the structural integrity and functional efficacy [1,2]. Traditionally, the detection and classification of such defects have relied heavily on manual inspection methods. Although widely used, these methods are subjective and significantly influenced by environmental variables, thus affecting the accuracy and reliability of defect detection [3,4]. Over time, there has been a notable shift towards automated systems that incorporate advancements in machine vision and machine learning to overcome the limitations of manual inspections [5]. In addition to providing accurate defect classification, automated systems offer benefits such as increased productivity, reduced labor costs, and improved safety in industrial environments [6]. Furthermore, advancements in sensor technology and data processing algorithms continue to drive innovation in automated defect detection systems, paving the way for more sophisticated and reliable solutions.

Recent advancements in machine vision technologies have propelled the field forward, revolutionizing defect detection and classification in the steel industry. High-resolution imaging systems, equipped with advanced sensors, offer unprecedented levels of detail and precision, allowing for accurate capture of surface irregularities and defects [7]. These imaging systems enable manufacturers to identify defects with greater clarity and sensitivity, leading to improved quality control measures and reduced production costs. Furthermore, real-time monitoring capabilities provide instantaneous feedback on the manufacturing process, allowing for timely interventions and adjustments to minimize defects and optimize production efficiency. Overall, these advancements in machine vision technology have significantly enhanced defect detection and classification in the steel industry, paving the way for more efficient and reliable manufacturing processes.

Deep learning technologies have offered substantial improvements by automating the feature extraction process, thereby enhancing the accuracy and efficiency of these systems [8,9,10]. Deep learning models, particularly Convolutional Neural Networks (CNNs), have transformed the landscape by enabling direct analysis of complex image data and eliminating labor-intensive manual feature extraction. Despite these advancements, deep learning approaches still face challenges, such as the need for extensive computational resources and large training datasets, which limit their feasibility in real-time industrial applications [11,12].

This study introduces a novel methodology that uniquely integrates multi-scale wavelet transforms with Recursive Feature Elimination (RFE) for the classification of steel surface defects. By leveraging the multi-scale analytical capabilities of wavelet transforms, our approach enables extraction of both broad and fine-grained textural features. Most representative features are then selected through a RFE process to effectively manage the unique characteristics of wavelet-transformed features from steel surface images. This dual enhancement significantly improves defect detection accuracy and operational efficiency, particularly in industrial settings where rapid and reliable defect classification is crucial.

## 2. Related Works

In this section, we survey the relevant literature to contextualize our study. We categorize the existing works into two main approaches: direct pixel-level image processing and wavelet-based signal analysis. This review provides insights into current methodologies and identifies areas for our research contribution.

### 2.1. Pixel-Level Image Processing

Recent innovations have markedly enhanced the accuracy and noise resilience of defect detection systems. Techniques such as Support Vector Machine (SVM) classifiers and artificial neural networks have shown significant improvements in defect detection accuracy [8,13]. Traditional methods employ various combinations of feature extraction and classification algorithms. For instance, the use of the Gray-Level Co-occurrence Matrix (GLCM), Gabor Wavelet, and Histogram of Oriented Gradients (HOG) has been prevalent in the detection of defects on steel surfaces [14]. These methods often integrate robust descriptors such as the Binary Gabor Pattern (BGP) and Principal Component Analysis (PCA) to reduce feature dimensionality and improve classification outcomes [13,15]. In [16], a new method was proposed that enhances the Canny operator with circle fitting and employs a median filter and Laplacian operator for preprocessing. They introduced an improved Canny operator with a probabilistic Hough transform for more accurate scratch detection on brake discs. Additionally, a combination of classifiers, such as the integration of SVM and Random Forest classifiers with Bayesian fusion, was proposed by [5]. This approach efficiently addresses complex defect characteristics and significantly outperforms traditional methods.

Deep learning has significantly advanced the field of defect detection by enabling autonomous feature extraction and reducing reliance on traditional labor-intensive methods. VGGs and Residual Networks (ResNets) have been effectively applied to datasets with limited samples and class imbalances by utilizing techniques such as transfer learning and image augmentation [17,18,19]. The advent of fully convolutional networks and end-to-end systems has notably increased classification accuracy [20]. Wan et al. [21] enhanced the VGG19 model with rapid image preprocessing and ROI image augmentation to handle limited sample sizes and imbalanced classes effectively. Similarly, Fu et al. [22] presented a SqueezeNet-based CNN optimized for steel surface defects, employing a Multi-Receptive Field (MRF) module to enhance detection across varying conditions. Further advancements in the field include Gao et al. [23], who introduced a semi-supervised learning approach using a CNN enhanced by Pseudo-Label (PL) to utilize unlabeled data prevalent in industrial settings. In another noteworthy study, Feng et al. [24] combined the RepVGG algorithm with a spatial attention mechanism, introducing the Xsteel Surface Defect Dataset (X-SDD) to focus on subtle defect characteristics. In [25], a defect detection method was proposed to address the scarcity of defective samples. This method combines a Frequency shift-convolutional Autoencoder (Fs-CAE) network with Statistical Process Control (SPC) thresholding. The Fs-CAE network preserves high-frequency information during image reconstruction and enhances defect detection accuracy, whereas incremental SPC thresholding enables automatic defect detection without labeled data during training. Moreover, Wang et al. [26] proposed an automatic defect detection network based on the Automated Sample Assignment (SCID) algorithm to balance positive and negative sample distributions automatically, addressing the issues of manual threshold setting and label assignment. Building on these developments, Wen et al. [27] further developed multiscale, multi-attention CNNs to improve defect classification by focusing computational resources on critical image areas. Despite these advancements, the extensive computational resources and large datasets required by these models pose challenges for their application in real-time systems [28,29].

### 2.2. Wavelet-Based Signal Analysis

Wavelet transform methods have also significantly contributed to the field, particularly in feature extraction for defect identification. The authors in [30] described a Content-Based Image Retrieval (CBIR) system using completed local binary patterns (CLBP) extracted from discrete wavelet transform (DWT) coefficients, optimizing the selection of mother wavelets and decomposition levels. In [31], the authors used an online corrosion monitoring system for carbon steel using wavelet transform analysis of electrochemical noise data, effectively identifying different corrosion types. Zheng et al. [32] used a legendre multi-wavelet transform combined with an auto-encoder, which represents a significant advancement, enhancing the discrimination of textural variations across defect types. These foundational methods have paved the way for our research, which incorporates a novel texture descriptor based on wavelet transforms, aiming to improve defect classification through a comprehensive analysis. Another recent work on defect detection proposed in [33] integrated wavelet theory and introduced a learnable M-band Wavelet Network (MWNet) for flexible signal representation. It dynamically focuses on the key components within each frequency band by using a sparsity constraint. The study in [34] further demonstrated the capability of wavelet transform in optical inspection tasks, where highlight suppression in texture-rich images was achieved by decomposing images into high- and low-frequency components. This approach preserves texture details while improving defect detection reliability, demonstrating the versatility of wavelet-based methods. Similarly, [35] proposed a CNN integrated with adaptive wavelet transform modules for texture classification. By analyzing spatial and frequency domain features in parallel, the method achieved superior performance on both medical ultrasound and natural texture datasets, highlighting the potential of combining wavelets with deep learning for enhanced classification accuracy. The authors in [36] introduce a wavelet sub and least mean square (LMS) adaptive filter to eliminate mechanical noise in rail health monitoring using acoustic emission. By transforming noisy signals and reference noise into wavelet coefficients and optimizing the filter parameters with a similarity coefficient metric, the proposed method outperforms conventional LMS and adaptive wavelet filtering methods. In [37], a Texture Defect Detection (TDD) algorithm that utilizes discrete wavelet frame decomposition to divide an image into several sub-bands was proposed. Thereafter, statistical features are extracted using a gray level co-occurrence matrix, and these features are applied to a support vector machine to classify defective images. Similarly, Kiswanto et al. [38] employed the Haar wavelet transform and GLCM for meat texture classification. Their method extracted statistical texture features, such as contrast, correlation, energy, homogeneity, and entropy, and it achieved high classification accuracy for fresh, frozen, and rotten meat using k-NN. This study underscores the effectiveness of wavelet-based feature extraction combined with statistical methods for texture classification, aligning with the goals of steel defect classification by capturing fine-grained and broad texture variations.

## 3. Proposed Method

**Figure 1:**
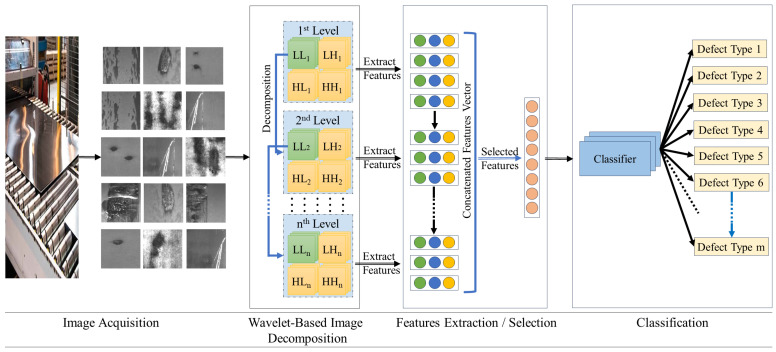
General scheme of the proposed WTD method for steel defect classification. The process involves image acquisition, wavelet-based image decomposition across multiple levels to extract both approximation (LL) and detail coefficients (LH, HL, HH), followed by feature extraction and selection. The selected features are then used to train a classifier to accurately identify various defect types.

In this study, we propose a novel method for classifying steel defects using a wavelet texture descriptor (WTD) for enhanced feature extraction. This methodology exploits the multiscale analytical capabilities of wavelet transforms, which are well-suited for analyzing steel surface images. By capturing essential spatial and frequency details, our method is adept at discerning subtle textural variations that are indicative of various defect types. This process involves wavelet decomposition, wherein the steel surface images are subjected to a sequence of low-pass and high-pass filters. This decomposition generates both approximation and detailed coefficients across multiple scales, which is essential for robust feature extraction. As depicted in Figure 1, our proposed method comprises three principal stages: (1) image decomposition using wavelet transform, (2) feature extraction through statistical and textural analysis, and (3) defect classification using a Support Vector Machine (SVM). Each stage is designed to build upon the preceding one, thereby ensuring a systematic and comprehensive approach to defect identification.

### 3.1. Wavelet-Based Image Decomposition

In our method, we examined the utilization of wavelet transforms for image processing, specifically focusing on their application in the analysis of steel surface images. Wavelet transforms are distinguished by their ability to perform multi-resolution analysis, which is essential for discerning both global architecture and intricate details within images. These transforms differ fundamentally from traditional Fourier transforms, which decompose signals into sinusoidal components but lack the capacity to localize these components spatially within the signal. Wavelet transforms mitigate this shortcoming by allowing for the analysis of images at multiple scales and resolutions, thereby facilitating simultaneous capture of spatial and frequency information.

Our method involves decomposing steel surface images using wavelet transforms, which are particularly advantageous for their ability to reveal textural details that are critical for detecting subtle variations indicative of defects. This process is carried out by applying a series of low-pass and high-pass filters and downsampling, thereby decomposing the image into approximation and detailed coefficients at multiple levels. Each level of decomposition isolates different scales of textural information, enabling a focused analysis of specific features essential for the subsequent stages of feature extraction and defect classification. This methodical breakdown is crucial for applications such as image compression and noise reduction, and it also plays a pivotal role in the robust textural feature extraction processes required for accurate defect classification in industrial settings.

#### 3.1.1. Wavelet Transform

In our experiments and method, we employ the Haar wavelet function, although numerous other wavelets exist, such as Daubechies and biorthogonal, among others. In particular, the Haar wavelet is well-suited for applications such as texture image analysis and steel defect detection because of its simplicity, computational efficiency, and effectiveness in capturing edge-like features that are common in such tasks. The Haar wavelet transform consists of two fundamental functions: scaling ϕ(t) (low-pass) and wavelet ψ(t) (high-pass) functions. These functions are applied in both the *x* and *y* directions to compute the detail and approximation coefficients, formally expressed as follows:

(1) $$\phi \left(t\right)=\left\{\begin{array}{cc} 1 & \mathrm{if}\;0\leq t<1\\ 0 & \mathrm{otherwise} \end{array}\right.$$

(2) $$\psi \left(t\right)=\left\{\begin{array}{cc} 1 & \mathrm{if}\;0\leq t<0.5\\ -1 & \mathrm{if}\;0.5\leq t<1\\ 0 & \mathrm{otherwise} \end{array}\right.$$

The first Equation (1) serves to approximate the signal at a coarser scale by averaging adjacent data points, whereas the second Equation (2) is crucial for capturing the differences between adjacent data points, effectively highlighting transitions or edges in the signal.

To process a 2D image, these functions result in the application of the following row and column filters:

- *Low-pass filter*: $\left[1,1\right]\times \frac{1}{\sqrt{2}}$
- *High-pass filter*: $\left[1,-1\right]\times \frac{1}{\sqrt{2}}$

The factor $\frac{1}{\sqrt{2}}$ serves as a normalization constant that ensures energy conservation and maintains the overall variance between the original and transformed signals.

This combination of simplicity in design and effectiveness in feature detection makes the Haar wavelet a preferred choice in various practical applications involving image processing.

#### 3.1.2. Transform Decomposition

**Figure 2:**
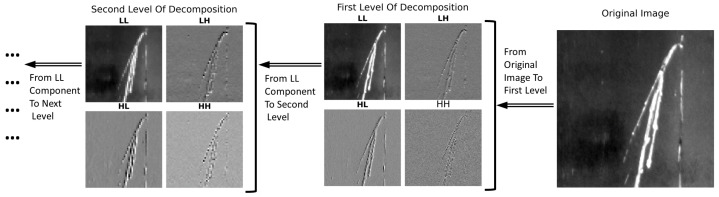
Illustration of the wavelet decomposition process using the Haar wavelet. The figure demonstrates the multi-level decomposition of an original image into its approximation (LL) and detail coefficients (LH, HL, HH) across two levels.

As shown in Figure 2, in the decomposition of the wavelet transform, an image is partitioned into four sub-bands, each representing distinct spatial frequency components: LL (low–low), LH (low–high), HL (high–low), and HH (high–high). Each sub-band is derived using combinations of low-pass and high-pass filters applied sequentially to the rows and columns of the image, followed by downsampling, which reduces the data size and focuses more on the essential frequencies. Hereafter is a concise explanation for the extraction of each sub-band:

LL (Approximation): This sub-band is computed by applying a low-pass filter both horizontally and vertically across the image, effectively isolating the low-frequency components that represent the overall structure or average content. This is based on the following formula:

(3) $$LL\left[i,j\right]=\frac{1}{2}\sum_{k,l}I\left[2i+k,2j+l\right]\cdot \phi \left[k\right]\cdot \phi \left[l\right]$$

where *I* is the original image, ϕ is the low-pass filter, and k,l are the convolution indices.

LH (horizontal detail): This sub-band highlights horizontal details by applying a low-pass filter to the rows and a high-pass filter to the columns. This is described by the following formula:

(4) $$LH\left[i,j\right]=\frac{1}{2}\sum_{k,l}I\left[2i+k,2j+l\right]\cdot \phi \left[k\right]\cdot \psi \left[l\right]$$

Here, ψ denotes the high-pass filter that captures the horizontal transitions and edges.

HL (vertical detail): Vertical details are emphasized in the HL sub-band through the application of a high-pass filter to rows and a low-pass filter to columns. It is defined by the following:

(5) $$HL\left[i,j\right]=\frac{1}{2}\sum_{k,l}I\left[2i+k,2j+l\right]\cdot \psi \left[k\right]\cdot \phi \left[l\right]$$

This formulation enhances the vertical structures and edges by filtering out horizontal low-frequency content.

HH (diagonal detail): Diagonal and finer details are isolated in the HH sub-band by applying high-pass filters both horizontally and vertically. It is defined as follows:

(6) $$HH\left[i,j\right]=\frac{1}{2}\sum_{k,l}I\left[2i+k,2j+l\right]\cdot \psi \left[k\right]\cdot \psi \left[l\right]$$

This captures high-frequency components in both dimensions, highlighting transitions that are neither horizontal nor vertical.

Each of these sub-bands extracts specific types of information from the original image, making wavelet decomposition a powerful tool for image analysis, compression, and feature extraction. The transformation allows for the separate processing of different frequency components, facilitating tasks such as low-level image analysis, noise reduction, and image enhancement based on the spatial characteristics of the content. To capture more details, we perform multi-level decomposition. The LL component from one level is used as the input for the next level, allowing for a deeper analysis of the low-frequency content of the image. This iterative process highlights finer details at each subsequent level, which is crucial for detecting subtle defects in textured surfaces such as steel.

### 3.2. Feature Extraction

Following the multi-level wavelet transforms of steel surface images into approximation and detail coefficients at each level, significant features are meticulously extracted for their discriminative power in distinguishing various defect types. This extraction is predicated on a rigorous statistical analysis of the wavelet coefficients across all decomposition levels to identify the features that best characterize the defects. After extracting these features, we perform feature selection and analysis using the Recursive Feature Elimination (RFE) algorithm. Post-selection, these features are normalized to mitigate scale disparities and enhance convergence during model training. This normalization is vital, as it ensures uniformity across the data set, preventing any single feature from skewing the classification results due to scale differences.

Approximation coefficients: At each wavelet decomposition level, the low-frequency approximation coefficients (LL) capture the general textural and brightness attributes of the image. For our analysis, we extract statistical features solely from the LL coefficients at the first level of decomposition. This approach minimizes feature redundancy and overfitting, thereby ensuring that the derived features effectively represent the key characteristics of the image without introducing excessive complexity. Specifically, we calculate mean μ, standard deviation σ, skewness γ, and kurtosis κ. The mean and variance provide insights into the central tendency and dispersion, respectively, whereas skewness and kurtosis reveal the asymmetry and tailedness of the pixel distribution. These statistics are fundamental for recognizing broad textural patterns that are potentially indicative of different defect types. Initially, these calculations are performed without normalization to maintain the original data scale and distribution. Mathematically, these features are computed as follows:

(7) $$\mu =\frac{1}{N}\sum_{i=1}^{N}x_{i}.$$

(8) $$\sigma^{2}=\frac{1}{N}\sum_{i=1}^{N}{\left(x_{i}-\mu \right)}^{2}.$$

(9) $$\gamma =\frac{\frac{1}{N}\sum_{i=1}^{N}{\left(x_{i}-\mu \right)}^{3}}{{\left(\frac{1}{N}\sum_{i=1}^{N}{\left(x_{i}-\mu \right)}^{2}\right)}^{3/2}}.$$

(10) $$\kappa =\frac{\frac{1}{N}\sum_{i=1}^{N}{\left(x_{i}-\mu \right)}^{4}}{{\left(\frac{1}{N}\sum_{i=1}^{N}{\left(x_{i}-\mu \right)}^{2}\right)}^{2}}.$$

Detail coefficients: From the coefficients —LH (horizontal detail), HL (vertical detail), and HH (diagonal detail)— extracted at every decomposition level, a comprehensive set of features is derived to capture finer textural and frequency variations. As with the approximation coefficients, we compute the mean, variance, skewness, and kurtosis for the detail coefficients. Using the Gray-Level Co-occurrence Matrix (GLCM) [39], we extract metrics such as contrast, dissimilarity, homogeneity, energy, and correlation. These properties provide a nuanced perspective by evaluating the spatial relationships between pixel intensities, which is critical for identifying subtle textural deviations associated with specific defect types. These features are defined as follows:

(11) $$\mathrm{Contrast}=\sum_{i,j}GLCM\left(i,j\right)\cdot {\left(i-j\right)}^{2}.$$

(12) $$\mathrm{Dissimilarity}=\sum_{i,j}GLCM\left(i,j\right)\cdot \left|i-j\right|.$$

(13) $$\mathrm{Homogeneity}=\sum_{i,j}\frac{GLCM(i,j)}{1+|i-j|}.$$

(14) $$\mathrm{Energy}=\sum_{i,j}GLCM{\left(i,j\right)}^{2}.$$

(15) $$\mathrm{Correlation}=\frac{\sum_{i,j}\left(i\cdot j\right)\cdot GLCM\left(i,j\right)-\mu_{x}\cdot \mu_{y}}{\sigma_{x}\cdot \sigma_{y}}.$$

where *N* represents the total number of pixels, $x_{i}$ denotes the intensity of the $i^{t}h$ pixel in the detail coefficients, and GLCM is the gray-level co-occurrence matrix, calculated as follows:

(16) $$GLCM\left(i,j\right)=\sum_{m=1}^{M}\sum_{n=1}^{N}\left\{\begin{array}{cc} 1, & \mathrm{if}\;I(m,n)=i\\ & \;\mathrm{and}\;I(m+\delta_{x},n+\delta_{y})=j\\ 0, & \mathrm{otherwise} \end{array}\right..$$

where *M* and *N* are the dimensions of the image, $\delta_{x}$ and $\delta_{y}$ represent the spatial offsets, and I(m,n) denotes the intensity of the pixel at position (m,n) in the image.

Additionally, we incorporate Local Binary Patterns (LBPs) [40,41] to capture the local spatial structure of the band by thresholding the neighborhood of each coefficient and encoding the result as a binary number. We focus on uniform patterns with at most two transitions between 0 and 1 in their binary representation, making them capable of capturing edges, corners, and spots. The formula for calculating the LBP value for a pixel is as follows:

(17) $${\mathrm{LBP}}_{P,R}=\sum_{p=0}^{P-1}s\left(g_{p}-g_{c}\right)\cdot 2^{p}$$

where:

- *P* is the number of neighbors.
- *R* is the radius of the neighborhood.
- $g_{c}$ is the gray value of the center coefficient.
- $g_{p}$ is the gray value of the *p*-th neighbor.
- s(x) is a thresholding function, defined as follows:

$$s\left(x\right)=\left\{\begin{array}{cc} 1 & \mathrm{if}\;x\geq \;0\\ 0 & \mathrm{if}\;x<0 \end{array}\right.$$

Furthermore, the power spectral density (PSD) is extracted, allowing for the computation of spectral energy $E_{\mathrm{spec}}$ and spectral entropy $H_{\mathrm{spec}}$. These measurements offer insight into the energy distribution and randomness within the frequency components, which are crucial for distinguishing between normal textures and anomalies. These features are as follows:

(18) $$E_{\mathrm{spec}}=\sum_{f}{\left|F\left(f\right)\right|}^{2}.$$

(19) $$H_{\mathrm{spec}}=-\sum_{f}{\left|F\left(f\right)\right|}^{2}\cdot {\log(|F\left(f\right)|}^{2}).$$

After extracting these features, we apply the Recursive Feature Elimination (RFE) algorithm for feature selection. It iteratively removes the least-important features based on their impact on model performance, ultimately resulting in a refined set of features optimized for classification.

Normalization: To ensure that features from all levels contribute equally to the classification models, we employ standardization:

(20) $$z=\frac{x-\mu }{\sigma }.$$

The normalized and selected feature set is then input into our classification models. This method leverages a rich and multiscale feature set to efficiently discern and classify various steel defects efficiently. By systematically analyzing both low-level and high-level properties derived from wavelet coefficients and ensuring that these features are properly normalized and integrated, our classification algorithms are furnished with an optimal representation of the data. This not only enhances the accuracy but also the robustness of defect classification, which is vital for industrial applications where detailed texture analysis is essential.

### 3.3. Classification

For the classification task, we employed a Support Vector Machine (SVM) owing to its effectiveness in handling high-dimensional data derived from wavelet features and its robustness to outliers. We utilized a radial basis function (RBF) kernel to manage the non-linear relationships between defect classes. To optimize the SVM model, we performed a grid search to fine-tune parameters such as the penalty parameter *C* and kernel coefficient γ, ensuring optimal model performance.

## 4. Experiments, Results, and Discussion

This section presents a comprehensive evaluation of the proposed Wavelet Texture Descriptor method for steel surface defect classification. The experiments are designed to assess the performance and robustness of our method through various analyses and comparisons against state-of-the-art methods. Additionally, an ablation study was conducted to investigate the impact of the different components of the proposed method.

### 4.1. Experiment Setup

This subsection describes the datasets, evaluation metrics, and configurations used to assess the performance of the proposed method for classifying steel surface defects.

**Figure 3:**
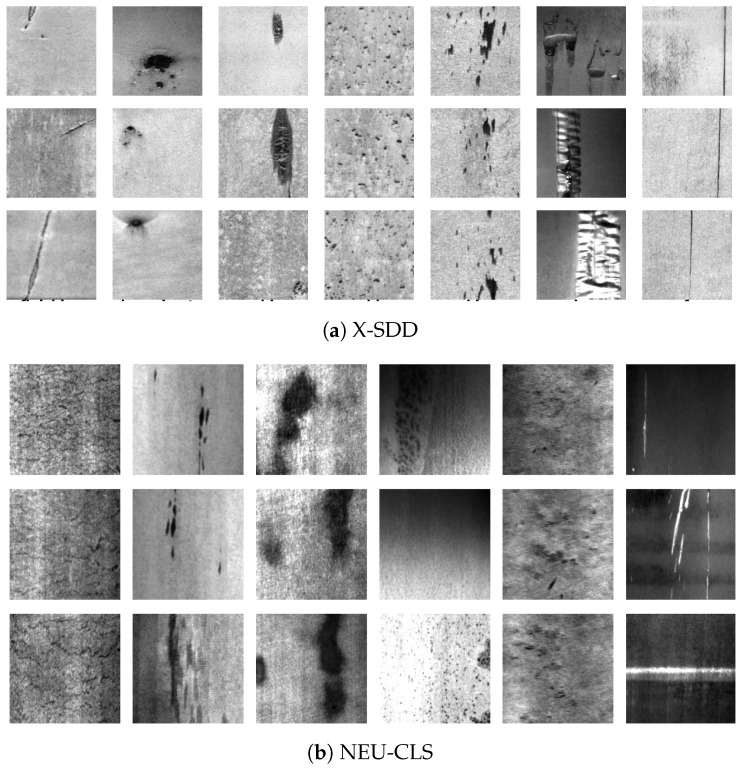
Representative samples from each defect class of the employed X-SDD and NEU-CLS datasets. (**a**) X-SDD: Classes from left to right are finishing roll printing, iron sheet ash, oxide scale of plate system, oxide scale of temperature system, red iron, slag inclusion, and surface scratch. (**b**) NEU-CLS: Classes shown from left to right are crazing, inclusions, patches, pitted surface, rolled-in scale, and scratches.

X-SDD The highly imbalanced Xsteel Surface Defect Dataset (X-SDD) [24] is comprised of 1360 images, each meticulously curated to enhance automated inspection systems for steel surfaces. These comprise 128×128 pixel images. Representative samples from this dataset are shown in Figure 3a. To circumvent the potential loss of information inherent in the downsampling operations, the images were resized to 256 × 256 pixels. This adjustment facilitates the extraction of more detailed features.

NEU-CLS is a balanced dataset that has been widely adopted in the steel industry for product inspection and defect analysis. It encompasses 1800 grayscale images that are evenly distributed across six typical defect categories of hot-rolled steel strips. These images were scanned at a resolution of 200 × 200 pixels in a grayscale format. Representative samples from each defect category are shown in Figure 3b. Owing to variations in illumination and material properties, the NEU-CLS dataset presents two significant challenges for defect classification: (1) high intra-class variability, where defects within the same category exhibit diverse appearances such as different orientations and shapes of scratches, and (2) substantial similarity among different defect classes, such as between rolled-in scale, crazing, and pitted surfaces, which complicates their distinct classification.

We opted for 10-fold cross-validation to ensure a comprehensive evaluation of the datasets lacking predefined training and validation sets and exhibiting unbalanced class distributions, thus minimizing potential biases from fixed train–test splits.

To assess the effectiveness of the model across various defect types in our multi-class classification tasks, we calculate the precision, recall, F1 score, and accuracy metrics. To ensure a balanced evaluation of both sensitivity and specificity, we utilize macro-averaged versions of these metrics, which are particularly useful in scenarios with unbalanced class distributions.

- Macro-precision: describes the average precision for each class, treating each class equally regardless of size.
  (21) $$P_{\mathrm{macro}}=\frac{1}{N}\sum_{i=1}^{N}\frac{TP_{i}}{TP_{i}+FP_{i}}.$$
- Macro-recall: Also known as macro-average sensitivity, macro-recall is the average recall obtained across all classes.
  (22) $$R_{\mathrm{macro}}=\frac{1}{N}\sum_{i=1}^{N}\frac{TP_{i}}{TP_{i}+FN_{i}}.$$
- The macro F1 score: is the harmonic mean of macro-precision and macro-recall.
  (23) $$F1_{\mathrm{macro}}=2\times \frac{P_{\mathrm{macro}}\times R_{\mathrm{macro}}}{P_{\mathrm{macro}}+R_{\mathrm{macro}}}.$$
- Accuracy: calculates the ratio of correctly predicted observations to the total observations.
  (24) $$\mathrm{Accuracy}=\frac{\sum_{i=1}^{N}TP_{i}+TN_{i}}{\mathrm{Total}\;\mathrm{number}\;\mathrm{of}\;\mathrm{samples}}.$$

where $TP_{i}$, $FP_{i}$, and $FN_{i}$ represent the true positives, false positives, and false negatives for each class *i*, respectively, and *N* is the number of classes.

An SVM with an RBF kernel was employed for the classification task. To optimize the model, we performed a grid search with the following parameter ranges:

C∈{0.1,1,10,100} and γ∈{0.001,0.01,0.1,scale}.

### 4.2. Evaluation and Comparison of the Proposed Method Against Related Works

In this subsection, we evaluate the performance of our proposed method against several recent state-of-the-art approaches, proposed for the same task of steel surface defect classification. As shown in Table 1, our method outperformed all other methods, including those based on DNN, across both datasets. The performance metrics, including precision and accuracy, are calculated as described in Formulas (21)–(24) in the experiment setup subsection. It is worth mentioning that some studies have demonstrated that handcrafted descriptors are more effective for fine-grained texture images, particularly stationary textures compared to those based on CNNs [42]. Despite the increased complexity and diversity of defects in the XSDD dataset, our Wavelet Texture Descriptor (WTD) method maintains high accuracy, affirming its capability to discern subtle defect patterns enhanced by robust feature extraction through wavelet transforms.

In contrast to deep learning-based methods, which often require extensive datasets and are prone to overfitting in low-data scenarios, our method focuses on extracting and utilizing the most significant features. This approach reduces noise, enhances generalizability, and maintains consistent performance on complex texture datasets. Moreover, unlike CNN-based methods that demand significant computational resources and extensive hyperparameter tuning, our method is computationally efficient and better suited for industrial applications. The superior performance of our method can be attributed to its systematic elimination of less-impactful features, ensuring classification decisions rely on the most relevant textural information. This not only mitigates overfitting but also improves generalizability across varied datasets. Additionally, our method effectively manages large feature sets and adapts to diverse data complexities, offering a robust and balanced solution ideal for industrial defect detection. Its flexibility and reliability under varied conditions make it a more versatile and scalable alternative to state-of-the-art methods, particularly for applications requiring rapid and accurate defect analysis.

**Figure 4:**
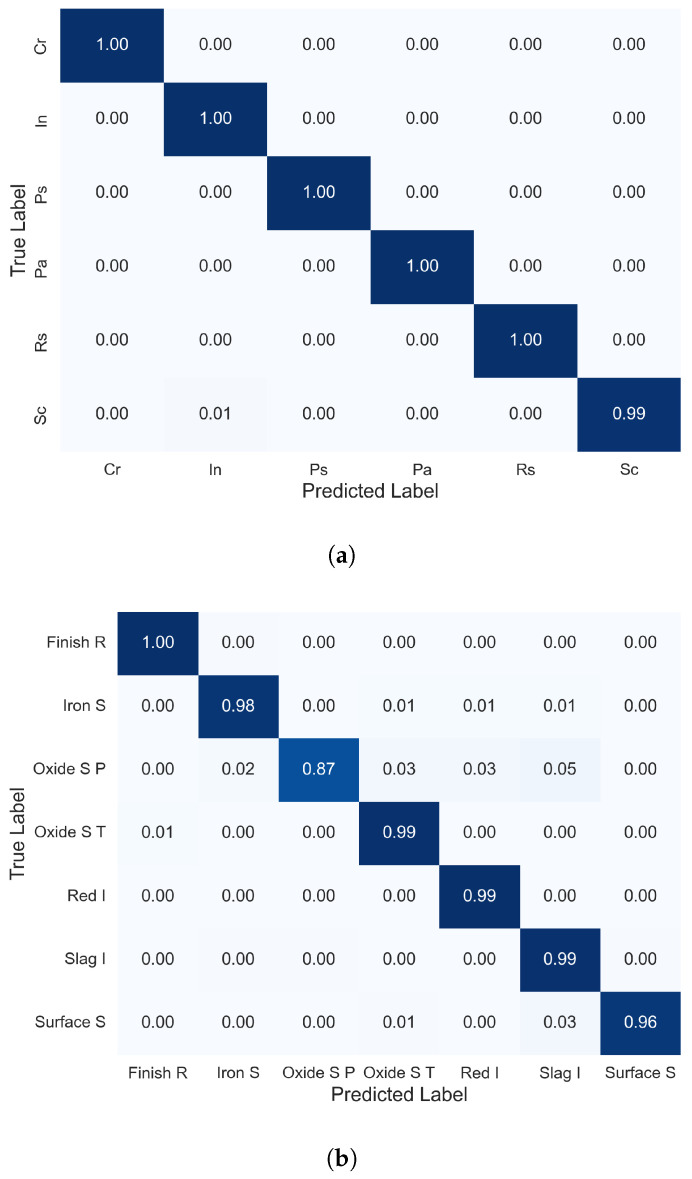
Confusion matrices illustrating the performance of the proposed method on each steel defect class for the datasets: (**a**) NEU_CLS and (**b**) X-SDD.

To further understand the performance of the proposed methods, we extracted and analyzed confusion matrices, as shown in Figure 4. Given the unbalanced nature of the X-SDD dataset, in which some defect types are more prevalent, normalizing the confusion matrix is essential. This normalization provides a fair comparison between classes by showing the proportion of correct and incorrect predictions relative to true class sizes. For the X-SDD dataset, classes such as “Finish R”, “Iron S”, “Oxide S T”, “Red I”, and “Slag I” exhibit near-perfect classification with normalized values close to 1.00, indicating excellent classifier performance. However, the “Oxide S P” class shows some misclassification, likely owing to overlapping features with other defect types. Despite this, the high diagonal values in the matrix indicate a strong overall performance, with only “Oxide S P” requiring further refinement. Similarly, the NEU-CLS dataset shows near-perfect classification for all classes except for some minor misclassifications in the “Sc” class, suggesting areas for improvement. However, the overall high diagonal values reflect the robustness and accuracy of the method.

In the subsequent subsections, we conduct an ablation study to evaluate the proposed WTD method. The experimental stages include wavelet selection analysis, feature selection and analysis, impact of decomposition levels, and evaluation with limited data. These stages collectively assess the effectiveness, robustness, and generalizability of the proposed method. The results and insights obtained from these experiments are discussed in detail in the following sections.

#### 4.2.1. Wavelet Selection Analysis

In our study, we investigated the impact of different wavelet transform techniques on steel defect classification. The utilization of multiscale analysis through various wavelets enables a thorough examination of the texture at multiple scales, which is pivotal for capturing the intricate details of surface defects. Wavelets such as Haar, Daubechies, biorthogonal, reverse biorthogonal, Symlets, and discrete Finite Impulse Response (FIR) each provide unique benefits in terms of edge detection and textural sensitivity. This diversity allows us to assess which wavelet optimally enhances classification accuracy by effectively distinguishing between subtle textural differences and abrupt changes characteristic of the different defect types. Through systematic testing, our method confirms that the choice of wavelet significantly influences the precision and reliability of defect classification, highlighting the importance of selecting an appropriate wavelet to improve diagnostic outcomes.

In this experiment, we utilized a straightforward feature representation combining variance and Gray Level Co-occurrence Matrix (GLCM) features such as dissimilarity and correlation to characterize steel surface defects. We employed a Support Vector Machine (SVM) classifier with 10-fold cross-validation to ensure the robustness of our results. The analysis was limited to the first level of wavelet decomposition, focusing on capturing the primary textural and structural features that are essential for initial defect identification. This setup allowed us to precisely evaluate the influence of different wavelets on the accuracy of defect classification across the datasets.

**Figure 5:**
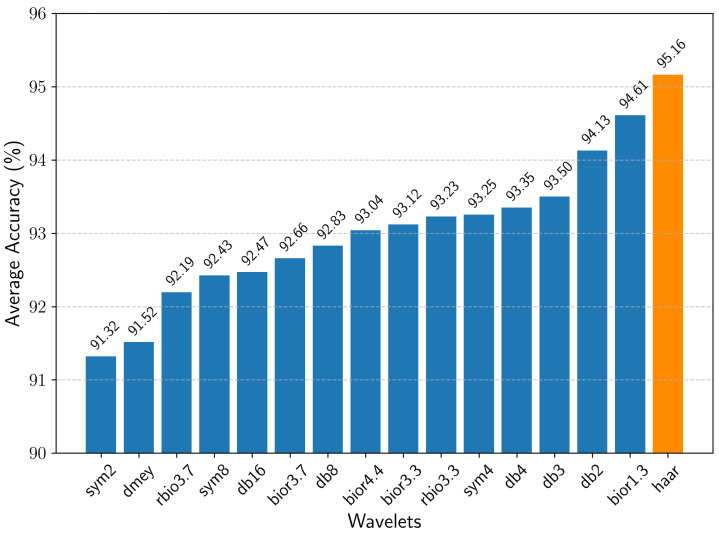
Average classification accuracy of wavelets across datasets, shown in ascending order. The Haar wavelet demonstrates the highest effectiveness for defect classification.

As shown in Figure 5, the average classification accuracy of different wavelets across datasets, arranged in ascending order, facilitates a direct comparison of their effectiveness. The data reveal that certain wavelets, notably Haar and Daubechies, yield higher accuracy, underscoring their effectiveness in capturing the essential features of defects under various conditions. The Haar wavelet achieves the highest average accuracy of 95.16%, closely followed by the biorthogonal (bior1.3) and Daubechies (db2) wavelets at 94.61% and 94.13%, respectively. This suggests that these wavelets, with their ability to handle abrupt changes in image textures, are particularly effective for the types of defects present in the datasets. However, wavelets such as discrete Meyer (dmey) and Symlet (sym2), while offering solid performance, do not reach the same level of accuracy, indicating potential limitations in scenarios requiring fine-grained detail detection.

Given the compelling evidence of its efficacy, we will continue to employ the Haar wavelet in subsequent phases of our research. This decision was based on its demonstrated capacity to effectively discern subtle textural differences and abrupt changes, which are critical for accurate defect classification.

#### 4.2.2. Feature Selection and Analysis

In this phase, we explored the impact of feature selection on the classification performance of steel surface defects using the Recursive Feature Elimination (RFE) algorithm. This method systematically identifies and eliminates less-important features and optimizes the performance of the model. RFE is particularly effective in high-dimensional datasets, helping to reduce overfitting and improve model generalizability. By iteratively training the model, removing the least-important feature, and retraining, we observe how the exclusion of each feature affects the overall accuracy. The following analysis provides a detailed account of feature importance for the NEU-CLS and X-SDD datasets, highlighting the critical features that contribute most significantly to accurate defect classification. The results of applying the RFE algorithm to the NEU-CLS dataset are presented in Table 2. This table lists the performance changes (Δ) in model accuracy after each iteration of feature removal.

The key observations from the analysis are as follows: The *mean* feature consistently demonstrated its importance in the early iterations. Its removal resulted in a small negative impact on the accuracy, indicating its significant role in classification. In contrast, the removal of *variance* showed a progressively increasing negative impact on accuracy, particularly in later iterations, highlighting its importance in maintaining the model performance. *Skewness* and *kurtosis* were among the features removed early in the process, suggesting that they were less critical than the other features in this dataset. *The correlation* feature had the most significant negative impact when removed, particularly in the final iterations, underscoring its crucial role in classification. Although it initially showed some negative impacts, *spectral entropy* was completely removed by iteration 7, indicating that it was less influential. Overall, the accuracy of the model improved slightly with the elimination of certain features, reaching a peak of 99.67%. This improvement indicates that some features contributed to noise rather than useful information, and their removal enhanced the overall performance of the model.

The RFE results highlight the importance of certain features, such as the mean, variance, and correlation, which are critical for accurate defect classification. The iterative elimination of less-important features not only simplifies the model but also enhances its generalization capability by reducing overfitting. The slight improvements in accuracy observed with the removal of non-essential features demonstrate the efficacy of the RFE approach in refining the feature set for better model performance.

#### 4.2.3. Impact of Decomposition Levels

In this subsection, we evaluated the impact of employing different levels of wavelet decomposition on the classification performance of the steel surface defect classification. By assessing both the individual and combined levels of decomposition, we aimed to understand how each level contributes to the overall model accuracy. This analysis considers both the individual performance at each level and the cumulative effect when combining all levels, helping to determine the optimal decomposition strategy for enhancing classification accuracy. Table 3 presents the results of this analysis.

As shown in Table 3, for NEU-CLS, the performance increased progressively with higher levels of decomposition, peaking at the fourth level with an accuracy of 98.94%. The combined levels achieved the highest accuracy of 99.67%, indicating that integrating multiple levels captures more comprehensive feature information. A similar trend is observed for the X-SDD dataset, with the performance improving significantly from the first to the fifth level. The highest individual-level accuracy was 97.43% at the fifth level, while the combined level reached 98.24%. As shown in the RFE Impact column, the removal of each level has a progressively negative effect, with the fifth level having the most significant negative impact ( −0.44% for NEU-CLS and −0. 96% for X-SDD). This suggests that higher levels of decomposition, especially the fifth level, contain critical information for classification and reinforce the importance of high-level decomposition features.

This integration leverages complementary information captured at different scales, leading to more robust and accurate classification models. The significant negative impact of removing higher levels, particularly the fifth level, underscores their critical role in feature extraction. These levels likely capture finer, more discriminative details that are essential for accurate defect classification.

By leveraging the complementary information captured at different scales, a comprehensive representation of the defect features is ensured, resulting in robust and accurate classification models. The significant negative impact of removing higher levels of decomposition, as shown in our RFE-based analysis, justifies incorporating multiscale wavelet features, which capture finer and more discriminative details crucial for precise defect classification.

#### 4.2.4. Evaluation with Limited Data

To assess the robustness of our method in scenarios with limited data, we conducted experiments using only 10 samples per class from each dataset. We extracted the first 10 samples from each class in both datasets, treating these subsets as separate datasets and using the same experimental setup. This evaluation is crucial for real-world industrial applications where collecting large datasets may not always be feasible. Our method achieved accuracies of 100% and 98.57% for the NEU-CLS and X-SDD datasets, respectively, demonstrating high efficiency in scenarios of data scarcity. This finding is particularly relevant for industrial applications, where resource constraints can limit the availability of large training datasets. By demonstrating high performance with fewer samples, our proposed method proved to be highly practical and effective for real-world deployments.

#### 4.2.5. Complexity and Computational Efficiency

The complexity of the proposed method for classifying steel defects using wavelet texture descriptors (WTD) and Support Vector Machines (SVMs) is primarily influenced by several stages: wavelet-based image decomposition, feature extraction, feature selection, and classification. The wavelet transform for an image of size N×N has a complexity of $O(N^{2}\log N)$ due to multi-level decomposition. Feature extraction and selection, involving statistical analysis and Recursive Feature Elimination (RFE), also have complexities around $O(N^{2})$ for extracting *F* features, and O(F·T·K) for RFE. The SVM training, particularly with a non-linear kernel like RBF, is the most computationally intensive, with a complexity of $K=O(M^{3}\cdot F)$, where *M* is the number of training samples. Therefore, the overall complexity can be approximated as $O(N^{2}\log N+M^{3}\cdot F)$, with the SVM training term being the dominant factor when *M* is large.

While deep learning methods are robust and capable of learning complex representations, they often require substantial computational resources throughout both training and testing phases, posing challenges in real-time processing environments where speed and resource efficiency are paramount. In contrast, our approach optimizes image representation to reduce complexity during application, leading to faster processing and more resource-efficient operations. In Table 4, we report the time consumed at each stage of our method. These experiments were conducted on an Intel Xeon(R) Platinum 8160M CPU 2.10 GHz × 48.

From the table, it is evident that our method demonstrates acceptable computation time, particularly during the classification (post-deployment) phase, which is crucial for rapid processing in industrial machinery. This efficiency ensures that the method can be integrated seamlessly into high-speed industrial environments where quick and reliable defect detection is essential. Similarly, the stages in the training phase do not require extensive processing time, with the exception of the feature extraction phase. This phase could be further optimized by selecting faster feature extraction methods to enhance overall efficiency.

While the WTD method demonstrates strong performance in defect classification, it has certain limitations. Its reliance on pre-defined wavelet types and decomposition levels may require tuning for different datasets. Additionally, the performance of the method may deteriorate with extremely high-dimensional data or severe inter-class similarities. The computational time required for feature extraction and Recursive Feature Elimination (RFE), especially with large datasets or high-resolution images, may pose a challenge for real-time applications. Furthermore, the effectiveness of the method is contingent upon the quality of the input image, as significant noise or poor resolution can impact classification accuracy. Future work could address these limitations by exploring adaptive wavelet selection, optimizing the RFE algorithm, and integrating WTD with deep learning to improve scalability and efficiency for diverse datasets and real-world applications.

## 5. Conclusions

In this paper, we presented a method based on wavelet transform and texture descriptors for the classification of steel surface defects. Our method significantly enhances the extraction of both broad and fine-grained textural features, which are crucial for accurate defect classification. It consistently outperformed state-of-the-art methods on both the NEU-CLS and X-SDD datasets, achieving state-of-the-art accuracy, precision, recall, and F1 score. Higher decomposition levels generally yield better performance by capturing more detailed features. The combined use of multiple decomposition levels is particularly beneficial, leading to superior classification accuracy. The extracted features from the wavelet texture descriptor (WTD) could be integrated with machine learning algorithms to enable scalable and automated defect detection systems. This approach has potential for improving real-time performance and scalability in industrial applications, representing a promising area for future exploration. For practical implementation, integrating our WTD method into existing industrial defect detection systems involves several challenges. These include optimizing the method for real-time processing, ensuring that it can handle large data volumes, maintaining accuracy under varying industrial conditions, ensuring compatibility with existing hardware and software, and considering implementation costs and ongoing maintenance requirements. Addressing these challenges is critical for successful deployment in real-world industrial applications. Future work will involve applying the WTD method in industrial environments to validate its robustness under practical conditions, such as variable image quality, hardware constraints, and diverse defect scenarios. Based on our findings, we suggest several potential directions for future research. These include developing real-time versions of our method, combining wavelet-based features with deep learning techniques, testing on more diverse datasets to ensure broader applicability, and creating methods for automatically selecting the optimal wavelet types and decomposition levels.

## Figures and Tables

**Table 1:** Model performance and comparison against related works on NEU-CLS and X-SDD datasets.

Dataset	Method	Accuracy	Precision	Recall	F1-Score
NEU-CLS	BRISK + SVM (2022) [43]	95.00			
	AECLPBP (2013) [11]	98.93			
	GCLBP (2018) [13]	99.11			
	Decaf (2019) [20]	99.27			
	ResNet34 (2019) [20]	99.33			
	VGG19+ (2023) [44]	97.20			
	RPNet (2024) [19]	98.73			
	VGG16 + CNN (2024) [45]	99.44			
	LWT + AE + SVM (2024) [32]	99.44			
	**WTD (Our)**	**99.67**	**99.68**	**99.67**	**99.67**
X-SDD	VGG16 (2021) [24]	92.65	91.70	90.46	90.92
	RepVGG + SA (2021) [24]	95.10	95.16	93.92	93.25
	ResNet50 + CBAM + FcaNet (2021) [46]	93.87	94.35	87.33	88.71
	LWT + AE + BPNN (2024) [32]	94.04	92.32	89.53	90.12
	LWT + AE + SVM (2024) [32]	95.37	94.45	92.90	93.33
	MobileNet-Pro (2024) [47]	96.47			
	**WTD (Our)**	**98.24**	**98.24**	**96.83**	**97.27**

**Table 2:** RFE-based feature importance and accuracy changes on NEU-CLS dataset.

	itr 1	itr 2	itr 3	itr 4	itr 5	itr 6	itr 7	itr 8	itr 9
Features	(Δ)	(Δ)	(Δ)	(Δ)	(Δ)	(Δ)	(Δ)	(Δ)	(Δ)
Mean	0.00	−0.06	−0.17	−0.17	−0.11	−0.17	−0.06	−0.22	−0.33
Variance	−0.05	−0.11	−0.11	−0.11	−0.06	−0.27	−0.17	−0.61	−0.39
Skewness	0.06	0.11	-	-	-	-	-	-	-
Kurtosis	0.06	0.06	−0.06	−0.06	0.00	0.11	-	-	-
Contrast	0.00	0.00	−0.06	−0.06	0.00	0.00	0.00	0.00	-
Dissimilarity	0.00	0.06	−0.06	−0.06	0.00	−0.11	−0.11	−0.11	−1.11
Homogeneity	−0.06	0.06	0.00	0.00	-	-	-	-	-
Energy	0.06	-	-	-	-	-	-	-	-
Correlation	−0.22	−0.11	−0.17	−0.17	−0.17	−0.17	−0.28	−1.16	−1.16
Lbp	0.00	−0.06	−0.06	−0.06	0.06	-	-	-	-
Spectral energy	0.00	0.00	0.00	-	-	-	-	-	-
Spectral entropy	−0.17	−0.17	−0.22	−0.22	−0.22	−0.22	0.00	-	-
**Combined features**	99.33	99.39	99.50	99.50	99.50	99.56	99.67	99.67	99.67

**Table 3:** Performance and RFE-based impact of decomposition levels on NEU-CLS and X-SDD datasets.

Level	Individual Accuracy		RFE Impact	
	NEU-CLS	X-SDD	NEU-CLS	X-SDD
1	87.50	74.56	−0. 06	−0. 15
2	95.06	87.43	−0. 11	−0. 22
3	97.89	93.90	−0. 22	−0. 37
4	98.94	96.10	−0. 17	−0. 29
5	98.72	97.43	−0. 44	−0. 96
**Combined Level**	99.67	98.24	-	-

**Table 4:** Average processing time in seconds for each stage of defect classification across datasets.

Stages	NEU-CLS	X-SDD	Average per Image
Preprocessing	0.3781	0.6023	0.0003
Feature extraction	752.8850	579.4701	0.4216
RFE	134.9992	65.3302	0.0634
Classification	0.7179	1.3500	0.0007

## Data Availability

The code is publicly available at https://github.com/dbelila/Wavelet-Texture-Descriptor-WTD (accessed on 19 October 2024).
